# Incidence of postoperative hypothermia and shivering and risk factors in patients undergoing malignant tumor surgery: a retrospective study

**DOI:** 10.1186/s12871-023-01991-8

**Published:** 2023-01-23

**Authors:** Rongrong Xu, Xinyi Hu, Zhirong Sun, Xuqin Zhu, Yonghong Tang

**Affiliations:** 1 Department of Anesthesiology, Fudan University Shanghai Cancer Center, 270 DongAn Road, Shanghai, 200032 China; 2 Department of Oncology, Shanghai Medical College, Fudan University, 270 DongAn Road, Shanghai, 200032 China; 3 Department of Nursing, Fudan University Shanghai Cancer Center, 270 DongAn Road, Shanghai, 200032 China

**Keywords:** Postoperative hypothermia, Shivering, Risk factors, Malignant tumor surgery

## Abstract

**Background:**

Perioperative hypothermia and shivering are common and can cause adverse outcomes. The aim of this study was to investigate the incidence of postoperative hypothermia and shivering and their risk factors in patients undergoing malignant tumor surgery.

**Methods:**

This retrospective study collected data from patients with American Society of Anesthesiologists physical status (ASA) I or II who underwent scheduled surgery from November 2020 to March 2021 at Fudan University Shanghai Cancer Center. Each patient’s core body temperature was measured at three time points: time point 1 (arrival at the postanesthesia care unit (PACU)), time point 2 (after 30-min care in the PACU), and time point 3 (at discharge from the PACU). At time point 1, if the patient’s body temperature was below 36 ℃, we provided an active forced-air warmer. At time point 2, if it was still below 36 ℃, the forced-air warmer was still applied until the patient was discharged from the PACU. If it reached 36 ℃, the forced-air warmer would be switched off. Univariate and multivariate logistic regression combined with stepwise methods and linear regression were used to explore risk factors for postoperative hypothermia and shivering.

**Results:**

The numbers (percentage) of 202 patients who developed postoperative hypothermia at the different time points were 52 (25.7%), 37 (18.3%) and 28 (13.9%). Eight patients (4.0%) experienced shivering. Multivariate logistic regression showed that high weight (OR = 0.923, 95% CI: 0.884 to 0.964, *P* = 0.0003) and low estimated blood loss (OR = 0.252, 95% CI: 0.115 to 0.550, *P* = 0.0005) were protective factors against hypothermia, while long surgical duration (OR = 3.339, 95% CI: 1.675 to 6.655, *P* = 0.0006) was an independent risk factor for hypothermia at time point 1. There was no risk factor associated with the occurrence of shivering (*P* > 0.05). There was a significant difference between the hypothermia and normothermia groups in the median length of stay in the PACU (59.0 vs. 49.0 min, *P* = 0.0123).

**Conclusions:**

Postoperative hypothermia occurred frequently. Weight, estimated blood loss and surgical duration were significantly associated with hypothermia on arrival at the PACU.

## Background

Perioperative hypothermia has been defined as a core body temperature below 36 ℃ during the perioperative period. It is a common clinical issue, occurring in up to 70% of noncardiac surgeries [1]. Hypothermia can increase the risks of certain adverse outcomes, such as surgical wound infection, blood loss and transfusion requirement, cardiac disorder and delayed discharge from the postanesthesia care unit (PACU) [2–4].

Shivering is also a common event closely related to body temperature during the postoperative period. Apart from causing an uncomfortable feeling and disrupting clinical monitoring, shivering may increase oxygen consumption by as much as 400% and may be associated with a higher risk of bleeding, adverse cardiovascular events, and other complications [5, 6].

In the past two decades, awareness of the mechanisms and risks associated with perioperative hypothermia has been increasing among anesthesiologists, nurses and surgeons. Thus, many medical specialty societies have provided guidelines for hypothermia prevention, which recommend monitoring the patient’s body temperature and providing active warming interventions throughout the perioperative period [7, 8]. However, despite the development of some effective active warming techniques, such as forced-air warming systems, self-warming mattresses, and irrigation and infusion of warmed fluids [9–12], a survey of 6 Asia–Pacific countries showed that compliance with international perioperative temperature management guidelines in the Asia–Pacific remains poor, especially in small hospitals [13].

The cause for hypothermia is likely iatrogenic due to the ambient operating room (OR) temperature, surgical exposure, infusion and irrigation of unwarmed fluids, anesthesia, and other factors [14]. Both general and neuraxial anesthesia greatly impair normal thermoregulatory control, thus resulting in hypothermia, especially for unwarmed surgical patients [15].

Surgical intervention is still the main treatment for solid tumors. Many patients with malignant tumors (MTs) are usually elderly, frail and have multiple comorbidities. As one previous study mentioned, elderly (> 65 years) patients and those with comorbidities were vulnerable to perioperative hypothermia [16]. Although some previous studies have been conducted regarding the risk factors of postoperative hypothermia, most of them covered all kinds of surgeries or anesthesia, including superficial, minor or major surgery and neuraxial or regional anesthesia [17–19]. As one of the largest cancer centers in China, almost every OR has been well equipped with monitoring and warming devices in our hospital in recent years. However, perioperative hypothermia still often occurs among the MT population in our daily clinical practice. Accordingly, we investigated the incidence of postoperative hypothermia and shivering and their risk factors focusing on relatively major and complicated abdominal, thoracic and spinal MT surgeries in recent years.

## Methods

This retrospective case‒control study was approved by the Ethics Committee of Fudan University Shanghai Cancer Center (FUSCC), China (protocol number: 2209260–4), and the requirement for informed consent was also waived by the Ethics Committee of FUSCC. As one previous study suggested [17], the surgery season had a specific effect on postoperative body temperature; therefore, we collected medical information of patients with American Society of Anesthesiologists physical status (ASA) I or II who underwent scheduled surgery in the cold weather (from November 2020 to March 2021) in FUSCC. Patients were excluded if they underwent emergent surgery or if their records of body temperature and shivering in the PACU were missing.

### Data collection

Our data were all obtained from the electronic database. We collected demographic and clinical data, including sex, age, height, weight, body mass index (BMI), baseline body temperature on the morning of surgery, intraoperative core body temperature, type of surgery (open or nonopen (laparoscopic and thoracoscopic) surgery), location of MT surgery, estimated blood loss, plasma transfusion, packed red blood cell (PRBC) transfusion, fluid intravenous infusion, type of anesthesia (general anesthesia (GA) or general anesthesia combined with epidural anesthesia (GEA)), duration of surgery, application of an infusion warming device (HOTLINE Fluid Warming Set) and an electrical self-warming mattress (ASTOPAD System), postoperative core body temperature, occurrence of shivering and length of stay (LOS) in the PACU.

### Body temperature monitoring

The intraoperative core body temperature was continuously measured through the nasal pharyngeal probe of the monitoring machine after intubation during the intraoperative period. However, which patient would receive active warming and body temperature monitoring was decided upon by the anesthesiologist’s discretion and habits.

In the PACU, the patient’s core body temperature was measured from the tympanic membrane using an infrared ear thermometer (Braun ThermoScan PRO 6000) at three time points: time point 1 (arrival at the PACU), time point 2 (after 30-min care in the PACU), and time point 3 (at discharge from the PACU).

Due to the limited acquisition of a forced-air warming device, our convention was to provide an active forced-air warmer in the cotton blanket, and the heating temperature was adjusted to 40 °C if the patient’s body temperature was below 36 °C immediately upon arrival at the PACU (time point 1). Otherwise, the patient was covered with an unheated cotton blanket if the body temperature was not below 36 ℃. At time point 2, nurses measured the patient’s body temperature again. If it was still below 36 °C, the forced-air warmer was still applied until the patient was discharged from the PACU. If it reached 36 ℃, the forced-air warmer would be switched off. If shivering occurred, patients would receive forced-air warming.

The core body temperature was below 35.5 ℃, which was defined as severe hypothermia. The room temperature in the PACU was maintained at 22–24 °C, while the humidity was maintained at 50–60% by the central control system.

### Statistical analysis

Sample size calculation was conducted in PASS software (Version 2021). Based on a previously reported incidence of hypothermia of approximately 56.7% [19] during the postoperative period with a 95% confidence level, 14% confidence interval width (two sided), and Z of 1.96, the calculated sample size was 117. Considering an approximately 10% drop-out rate, a total sample size of 130 patients was needed. In this study, we collected data from 202 patients during cold weather (from November to March).

Statistical analyses were performed in SAS (version 9.4; Cary, NC, USA). Continuous variables are presented as the mean ± standard deviation (SD) or median with interquartile range (IQR) and were compared using the independent Student’s t test or Wilcoxon Mann‒Whitney tests depending on the results of the normality test. Categorical variables are presented as numbers (percentage) and were compared using the Pearson chi-square test or Fisher’s exact test as appropriate.

Univariate and multivariate logistic regression combined with stepwise methods and linear regression were used to explore risk factors for postoperative hypothermia and shivering. The results were expressed as odds ratios (ORs) with 95% confidence intervals (CIs). *P* < 0.05 was considered statistically significant.

## Results

### Baseline characteristics of the study population

A total of 210 patients who underwent MT surgery were evaluated in this study. Eight patients were excluded due to emergent surgery or a lack of records of body temperature and shivering in the PACU. Ultimately, 202 patients were subjected to statistical analysis. Patients underwent abdominal, thoracic and spinal surgeries that included pancreatic, duodenal, cholecystic, hepatic, gastric, colorectal, gynecological, urinary, and esophageal MT procedures (Table 1).

**Table 1:** Comparisons of characteristics between the hypothermia and normothermia groups

Characteristics	Hypothermia (n = 52)	Normothermia (n = 150)	*P*
Sex, n (%)			0.494
Male	35 (67.3)	93 (62.0)	
Female	17 (32.7)	57 ((38.0)	
Age (years), M ± SD, n (%)	61.9 ± 10.3	58.0 ± 11.9	0.038
≤ 65	29 (55.8)	101 (67.3)	0.134
> 65	23 (44.2)	49 (32.7)	
Height (m), M ± SD	1.7 ± 0.1	1.7 ± 0.1	0.555
Weight (kg), M ± SD	60.1 ± 9.0	65.2 ± 9.9	0.001
BMI (kg/m^2^), M ± SD, n (%)	22.2 ± 3.3	23.7 ± 2.7	0.001
≥ 25	12 (23.1)	51 (34.0)	0.143
< 25	40 (76.9)	99 (66.0)	
Type of surgery, n (%)			0.169
Open surgery	31 (59.6)	105 (70.0)	
Nonopen (laparoscopic and thoracoscopic surgery)	21 (40.4)	45 (30.0)	
Estimated blood loss (ml), median (IQR), n (%)	100.0 (100.0)	130.0 (200.0)	0.472
≤ 200	48 (92.3)	109 (72.7)	0.023
200 > V ≤ 500	2 (3.85)	20 (13.3)	
500 < V ≤ 1000	2 (3.85)	12 (8.0)	
> 1000	0 (0.0)	9 (6.0)	
Plasma transfusion, n (%)			0.612
Yes	3 (5.8)	14 (9.3)	
No	49 (94.2)	136 (90.7)	
PRBC transfusion, n (%)			0.947
Yes	3 (5.8)	11 (7.3)	
No	49 (94.2)	139 (92.7)	
Liquid infusion (ml), median (IQR), n (%)	2100.0 (400.0)	2100.0 (500.0)	0.660
1000 < V ≤ 2000	20 (38.5)	71 (47.3)	0.268
> 2000	32 (61.5)	79 (52.7)	
Type of anesthesia, n (%)			0.106
GA	9 (17.3)	43 (28.7)	
GEA	43 (82.7)	107 (71.3)	
Duration of surgery (min), M ± SD, n (%)	196.9 ± 70.1	175.2 ± 76.8	0.074
≤ 120	4 (7.7)	36 (24.0)	0.039
120 < D ≤ 240	39 (75.0)	91 (60.7)	
> 240	9 (17.3)	23 (15.3)	
Application of warmed fluid infusion, n (%)			0.277
Yes	50 (96.2)	135 (90.0)	
No	2 (3.8)	15 (10.0)	
Application of a warming mattress, n (%)			0.322
Yes	0 (0.0)	6 (4.0)	
No	52 (100.0)	144 (96.0)	
Baseline body temperature in the morning (℃), M ± SD	36.7 ± 0.3	36.7 ± 0.3	0.861
Location of MT surgery, n (%)			0.003
Pancreatic and duodenal MT	4 (7.7)	25 (16.67)	
Hepatic and cholecystic MT	2 (3.8)	22 (14.67)	
Gastric MT	4 (7.7)	13 (8.67)	
Colorectal MT	13 (25)	36 (24)	
Gynecological MT	2 (3.8)	15 (10)	
Spinal MT	0 (0)	4 (2.67)	
Esophageal MT	24 (46.2)	25 (16.67)	
Urinary MT	3 (5.8)	10 (6.67)	

The hypothermia and normothermia groups were on arrival at PACU

*M* ± *SD* Mean ± standard deviation, *IQR* Interquartile range, *BMI* Body mass index, *PRBC* Packed red blood cell, *GA* General anesthesia, *GEA* General anesthesia combined with epidural anesthesia, *V* Volume, *D* Duration, *PACU* Postanesthesia care unit, *MT* Malignant tumor

The numbers (percentage) of patients who developed postoperative hypothermia at the three time points were 52 (25.7%), 37 (18.3%) and 28 (13.9%) immediately upon arrival at the PACU, after staying in the PACU for 30 min and at discharge from the PACU, respectively. Only 4 patients suffered from severe postoperative hypothermia. The number (percentage) of patients experiencing shivering was 8 (4.0%), and the shivering was relieved by active forced-air warming without any medical therapy.

The numbers of patients with normothermia at time point 1 who developed new hypothermia were 8 and 5 at time point 2 and 3, respectively. Among the patients with normothermia at time point 1 and 2, 2 of them developed new hypothermia at time point 3. Among the patients with hypothermia at time point 1 and normothermia at time point 2, 3 of them developed new hypothermia at time point 3. The core body temperature ranged from 35.7 ℃ to 35.9 ℃ among these new postoperative hypothermia patients.

Comparisons of demographic and clinical characteristics between the hypothermia and normothermia groups at time point 1 are shown in Table 1. Age, weight, BMI, estimated blood loss, surgical duration and location of MT surgery all showed statistically significant differences between the two groups (*P* < 0.05).

Only 30 patients received intraoperative temperature monitoring, and 20 of them developed intraoperative hypothermia and were not subjected to further statistical analysis. The intraoperative core body temperature ranged from 33.8 ℃ to 36.9 ℃ among these patients.

### Risk factors associated with hypothermia on arrival at the PACU

According to the univariate logistic regression, age, weight, BMI and estimated blood loss were associated with hypothermia at time point 1 (*P* < 0.05) (Table 2). Multivariate logistic regression showed that high weight (OR = 0.923, 95% CI: 0.884 to 0.964, *P* = 0.0003) and low estimated blood loss (OR = 0.252, 95% CI: 0.115 to 0.550, *P* = 0.0005) were protective factors against hypothermia, while long surgical duration (OR = 3.339, 95% CI: 1.675 to 6.655, *P* = 0.0006) was an independent risk factor for hypothermia at time point 1 (Table 2). Multivariate logistic regression also showed that there was no risk factor associated with severe postoperative hypothermia at time point 1.

**Table 2:** Risk factors associated with postoperative hypothermia on arrival at the PACU

Risk factors	Univariate logistic regression		Multivariate logistic regression	
	**Odds ratio (95% CI)**	***P***	**Odds ratio (95% CI)**	***P***
Sex	1.262 (0.648–2.458)	0.4941		
Male				
Female				
Age (years)	1.032 (1.001–1.063)	0.0400		
≤ 65	1.635 (0.858–3.116)	0.1352		
> 65				
Height (m)	0.987 (0.945–1.031)	0.5533		
Weight (kg)	0.941 (0.906–0.977)	0.0016	0.923 (0.884–0.964)	0.0003
BMI (kg/m^2^)	0.831 (0.739–0.935)	0.0021		
≥ 25	1.717 (0.829–3.557)	0.1456		
< 25				
Type of surgery	1.581 (0.821–3.043)	0.1705		
Open surgery				
Nonopen (laparoscopic and thoracoscopic) surgery				
Estimated blood loss (ml)	0.999 (0.997–1.014)	0.0530		
≤ 200	0.410 (0.204–0.824)	0.0124	0.252 (0.115–0.550)	0.0005
200 > V ≤ 500				
500 < V ≤ 1000				
> 1000				
Plasma transfusion	0.595 (0.204–0.824)	0.4295		
Yes				
No				
PRBC transfusion	0.774 (0.164–2.158)	0.7027		
Yes				
No				
Liquid infusion (ml)	1.000 (0.207–2.889)	0.9177		
1000 < V ≤ 2000	1.252 (0.607–2.584)	0.2690		
> 2000				
Type of anesthesia	1.920 (0.755–2.738)	0.1104		
GA				
GEA				
Duration of surgery (min)	1.000 (0.862–4.277)	0.3530		
≤ 120	1.689 (0.981–2.908)	0.0588	3.339 (1.675–6.655)	0.0006
120 < D ≤ 240				
> 240				
Application of warmed fluid infusion	3.638 (0.786–16.834)	0.0985		
Yes				
No				
Application of a warming mattress		0.9816		
Yes				
No				
Baseline body temperature in the morning (℃)	0.916 (0.344–2.439)	0.8602		

*BMI* Body mass index, *PRBC* Packed red blood cell, *GA* General anesthesia, *GEA* General anesthesia combined with epidural anesthesia, *V* Volume, *D* Duration, *PACU* Postanesthesia care unit, *95% CI* 95% confidence interval

### Risk factors associated with hypothermia at time point 2 (after 30-min care in the PACU)

According to the univariate logistic regression, weight, BMI, estimated blood loss and type of anesthesia were associated with hypothermia at time point 2 (*P* < 0.05) (Table 3). Multivariate logistic regression showed that high weight (OR = 0.917, 95% CI: 0.872 to 0.964, *P* = 0.0007) and low estimated blood loss (OR = 0.273, 95% CI: 0.110 to 0.674, *P* = 0.0049) were protective factors against hypothermia, while long surgical duration (OR = 2.703, 95% CI: 1.299 to 5.623, *P* = 0.0078) was an independent risk factor for hypothermia at time point 2 (Table 3).

**Table 3:** Risk factors associated with postoperative hypothermia after 30-min care in the PACU

Risk factors	Univariate logistic regression		Multivariate logistic regression	
	**Odds ratio (95% CI)**	***P***	**Odds ratio (95% CI)**	***P***
Sex	1.254 (0.588–2.673)	0.5578		
Male				
Female				
Age (years)	1.032 (0.997–1.068)	0.0728		
≤ 65	1.947 (0.946–4.009)	0.0704		
> 65				
Height (m)	0.986 **(**0.939**–**1.036)	0.5763		
Weight (kg)	0.934 (0.894–0.976)	0.0022	0.917 (0.872–0.964)	0.0007
BMI (kg/m^2^)	0.813 (0.712–0.929)	0.0024		
≥ 25	1.812 (0.777–4.228)	0.1688		
< 25				
Type of surgery	2.012 (0.972–4.166)	0.0597		
Open surgery				
Nonopen (laparoscopic and thoracoscopic) surgery				
Estimated blood loss (ml)	0.998 (0.997–1.000)	0.0920		
≤ 200	0.411 (0.177–0.954)	0.0384	0.273 (0.110–0.674)	0.0049
200 > V ≤ 500				
500 < V ≤ 1000				
> 1000				
Plasma transfusion	1.418 (0.435–4.623)	0.5628		
Yes				
No				
PRBC transfusion	1.235 (0.327–4.669)	0.7554		
Yes				
No				
Liquid infusion (ml)	1.000 (0.999–1.001)	0.7915		
1000 < V ≤ 2000	1.252 (0.607–2.584)	0.5424		
> 2000				
Type of anesthesia	3.383 (1.137–10.069)	0.0285		
GA				
GEA				
Duration of surgery (min)	1.000 (1.000–1.000)	0.4925		
≤ 120	1.522 (0.830–2.791)	0.1748	2.703 (1.299–5.623)	0.0078
120 < D ≤ 240				
> 240				
Application of warmed fluid infusion	6.484 (0.746–56.323)	0.0901		
Yes				
No				
Application of a warming mattress		0.9842		
Yes				
No				
Baseline body temperature in the morning (℃)	0.421 (0.132–1.343)	0.1437		

*BMI* Body mass index, *PRBC* Packed red blood cell, *GA* General anesthesia, *GEA* General anesthesia combined with epidural anesthesia, *V* Volume, *D* Duration, *PACU* Postanesthesia care unit, *95% CI* 95% confidence interval

### Risk factors associated with hypothermia at time point 3 (at discharge from the PACU)

According to the univariate logistic regression, age, weight, BMI, type of surgery and baseline body temperature on the morning of surgery were associated with hypothermia at time point 3 (*P* < 0.05) (Table 4). Multivariate logistic regression showed that high BMI (OR = 0.760, 95% CI: 0.648 to 0.892, *P* = 0.0008) and high baseline body temperature on the morning of surgery (OR = 0.087, 95% CI: 0.018 to 0.418, *P* = 0.0023) were protective factors against hypothermia, while advanced age (OR = 3.470, 95% CI: 1.398 to 8.612, *P* = 0.0073) and nonopen (laparoscopic and thoracoscopic) surgery (OR = 5.263, 95% CI: 1.994 to 13.887, *P* = 0.0008) were independent risk factors for hypothermia at time point 3 (Table 4).

**Table 4:** Risk factors associated with postoperative hypothermia at discharge from the PACU

Risk factors	Univariate logistic regression		Multivariate logistic regression	
	**Odds ratio (95% CI)**	***P***	**Odds ratio (95% CI)**	***P***
Sex	1.527 (0.637–3.664)	0.3429		
Male				
Female				
Age (years)	1.035 (0.996–1.076)	0.0821		
≤ 65	2.810 (1.246–6.336)	0.0128	3.470 (1.398–8.612)	0.0073
> 65				
Height (m)	0.992 (0.939–1.048)	0.7656		
Weight (kg)	0.951 (0.908–0.996)	0.0317		
BMI (kg/m^2^)	0.839 (0.727–0.969)	0.0171	0.760 (0.648–0.892)	0.0008
≥ 25	1.423 (0.572–3.545)	0.4482		
< 25				
Type of surgery	2.783 (1.236–6.263)	0.0134	5.263 (1.994–13.887)	0.0008
Open surgery				
Nonopen (laparoscopic and thoracoscopic) surgery				
Estimated blood loss (ml)	0.997 (0.995–1.000)	0.0809		
≤ 200	0.311 (0.092–1.047)	0.0594		
200 > V ≤ 500				
500 < V ≤ 1000				
> 1000				
Plasma transfusion	2.064 (0.622–6.852)	0.2365		
Yes				
No				
PRBC transfusion	1.778 (0.464–6.820)	0.4013		
Yes				
No				
Liquid infusion (ml)	1.000 (0.999–1.001)	0.7304		
1000 < V ≤ 2000	1.315 (0.582–2.971)	0.5098		
> 2000				
Type of anesthesia	1.702 (0.612–4.737)	0.3082		
GA				
GEA				
Duration of surgery (min)	1.000 (1.000–1.000)	0.4950		
≤ 120	1.441 (0.733–2.830)	0.2894		
120 < D ≤ 240				
> 240				
Application of warmed fluid infusion	2.098 (0.381–11.551)	0.3944		
Yes				
No				
Application of a warming mattress		0.9788		
Yes				
No				
Baseline body temperature in the morning (℃)	0.185 (0.047–0.727)	0.0157	0.087 (0.018–0.418)	0.0023

*BMI* Body mass index, *PRBC* Packed red blood cell, *GA* General anesthesia, *GEA* General anesthesia combined with epidural anesthesia, *V* Volume, *D* Duration, *PACU* Postanesthesia care unit, *95% CI* 95% confidence interval

### Risk factors associated with shivering

Univariate and multivariate logistic regression showed that there was no risk factor associated with the occurrence of shivering (*P* > 0.05). Multivariate linear regression showed that sex and age were slightly related to the duration of shivering (*P* = 0.0513).

### Clinical outcome of hypothermia

The median LOS in the PACU in the hypothermia and normothermia groups was 59.0 and 49.0 min, respectively, and there was a significant difference between the two groups (*P* = 0.0123) (Table 5).

**Table 5:** Length of the PACU stay of patients with or without postoperative hypothermia

**Variable**	**Total** (n = 202)	**Hypothermia** (n = 52)	**Normothermia** (n = 150)	***P***
LOS in the PACU (min), median (IQR)	51.5 (33.5)	59.0 (37.5)	49.0 (28.5)	0.0123

*PACU* Postanesthesia care unit, *LOS* Length of stay, *IQR* Interquartile range

## Discussion

In this study, the incidence of postoperative hypothermia was 25.7% in patients immediately upon arrival at the PACU. After active forced-air warming for patients with hypothermia, the incidence of hypothermia was 13.9% at discharge from the PACU; to some extent, active forced-air warming might be an effective method to rectify hypothermia. In addition, only 8 patients experienced shivering in the PACU. High weight and BMI, high baseline body temperature on the morning of surgery and low estimated blood loss were protective factors for postoperative hypothermia, while advanced age, nonopen (laparoscopic and thoracoscopic) surgery, and long duration of surgery were risk factors for postoperative hypothermia.

The previously reported incidence of postoperative hypothermia in the PACU varied widely, ranging from 4% to 56.7% [19, 20], probably in part because of differences in patients and surgeries, which were included in these studies. Previous studies suggested that patients undergoing major-plus or nonsuperficial surgery were vulnerable to perioperative hypothermia [16]. Therefore, we paid closer attention to these MT populations undergoing relatively major and complicated abdominal, thoracic and spinal surgeries and excluded those patients undergoing endoscopic or superficial surgeries, such as breast and thyroid surgeries. In our study, the incidence of postoperative hypothermia was 25.7% on arrival at the PACU, which was similar to the incidence in one previous study [17].

We found that high body weight and BMI ≥ 25 were protective factors against postoperative hypothermia, which was consistent with previous findings [17, 21]. A total of 202 patients were included in our study, and 68.8% of the patients’ BMIs were < 25, perhaps because most MT patients were frail with poor nutritional status. An age older than 65 years was another risk factor for postoperative hypothermia, as suggested in previous studies [16, 17]. Elderly patients, particularly those who are over 65 years old, have less effective regulatory ability of their central nervous system and an increased susceptibility to hypothermia [9]. As mentioned above, many MT patients possess risk factors for hypothermia, including poor nutritional status, low BMI and advanced age; thus, it is necessary for us to take active measures to prevent hypothermia in this population.

We also found that high baseline body temperature on the morning of surgery could effectively protect patients from hypothermia, which was consistent with previous studies [17, 21, 22]. Thus, raising the baseline body temperature by active prewarming should be an effective strategy to prevent perioperative hypothermia. Several studies have also suggested that prewarming with a forced-air warming system is an effective, simple and convenient method to prevent hypothermia [23]. However, most forced-air warming systems need to be purchased at patients’ own expense because of the reimbursement policy in China, which might be a barrier to generalizing these devices to more patients.

It was not surprising that a long duration of surgery was a risk factor for hypothermia, which was also consistent with some previous reports [21, 22]. A longer duration of surgery increased the time that the patient was exposed to the ambient environment and resulted in a longer duration of anesthesia and the receipt of more unwarmed CO_2_ in the laparoscopic or thoracoscopic surgery. In this study, low estimated blood loss was associated with decreased postoperative hypothermia, which was consistent with one prior study of advanced ovarian cancer surgery [24]. However, another study indicated that blood loss was not associated with hypothermia. This inconsistency might be due to the different volumes of blood loss in different studies [17, 22].

With regard to the type of surgery, some previous studies suggested that laparoscopic surgery was not associated with a higher incidence of perioperative hypothermia than open surgery [25, 26]. However, we found that nonopen surgery (laparoscopic and thoracoscopic surgery) increased the incidence of postoperative hypothermia, which was similar to the finding in one previous study [17]. A large amount of cold, dry CO_2_ was continuously insufflated into the body during the laparoscopic or thoracoscopic surgery, which then could cause heat loss and a lower body temperature. A meta-analysis also revealed that during laparoscopic abdominal surgery, the core body temperature was significantly lower in the cold CO_2_ groups than in the heated, humidified CO_2_ groups [27]. Therefore, as one previous study indicated, perhaps the use of warm and humidified CO_2_ for peritoneal insufflation was a safe, feasible and cost-effective intervention to prevent hypothermia [28].

After 30 min of care in the PACU, we found that GEA, one type of anesthesia, was associated with increased postoperative hypothermia in univariate logistic regression. However, GEA was not significantly associated with postoperative hypothermia in multivariate logistic regression, perhaps because of the small sample size of only 52 patients in the GA group. In our hospital, we often perform epidural anesthesia during general anesthesia for abdominal and thoracic surgeries. One previous retrospective study also demonstrated that epidural anesthesia was associated with intraoperative hypothermia in advanced ovarian cancer surgery [24]. The mechanism by which epidural anesthesia results in hypothermia might involve impairing central thermoregulatory control and preventing vasoconstriction and shivering in blocked areas [15]. Therefore, further randomized controlled studies are required to establish the precise relationship between epidural anesthesia and hypothermia in MT surgery.

In this study, only a total of 8 patients experienced shivering, which was less than the numbers of patients reported in previous studies [21, 29]. However, the core body temperatures of patients with shivering were not all below 36 ℃. Shivering traditionally is attributed to hypothermia, but it is not always thermoregulatory. As a previous study recommended, shivering should be treated by active warming [30]. In fact, shivering disappeared in almost all the patients by active forced-air warming without supplementary medical therapy in our study. To a certain extent, we found that advanced age and male sex showed little association with a longer duration of shivering. Thus, the relationship between shivering and age and sex can be investigated in further prospective studies.

There were some limitations in this study. First, only 30 patients received intraoperative temperature monitoring, perhaps because of lack of awareness of perioperative temperature management among many anesthesiologists. Thus, it limited possible findings of this study, and we could not determine the incidence of intraoperative hypothermia and the accurate relationship between postoperative hypothermia and intraoperative hypothermia. We will pay more attention to perioperative temperature management and investigate the incidence of intraoperative hypothermia and their risk factors in patients undergoing MT surgery in the future study. Second, we did not record the ambient temperature in the OR and PACU, even though there was a monitoring panel in the OR. Because there was no temperature monitoring in the PACU, we could not record the room temperature in the PACU. However, the room temperature in the OR and PACU was controlled by the central control system according to the specified standard in our hospital. Third, statistical analysis of risk factors associated with hypothermia at time point 2 and 3 was based on the facts that hypothermia was rectified by active forced-air warming, and the results of the two time points might be not as accurate as the time point 1. However, to some extent, these results also helped us acquire potential risk factors related to hypothermia for future studies in more detail.

## Conclusions

This study demonstrated that high weight and BMI, high baseline body temperature on the morning of surgery and low estimated blood loss were associated with a decreased risk of postoperative hypothermia, while advanced age, laparoscopic and thoracoscopic surgery and long duration of surgery were associated with an increased risk of postoperative hypothermia. The incidence of postoperative hypothermia was not low, and patients with hypothermia stayed in the PACU longer than those with normothermia. In our study, most patients did not receive temperature monitoring in the intraoperative period, which reflected the finding that many anesthesiologists still neglected temperature management. Therefore, we need to raise awareness and follow the recommendations of guidelines on the prevention of perioperative hypothermia.

## Data Availability

The datasets used and/or analyzed during the current study are available from the corresponding author on reasonable request.

## Abbreviations

ASA: American Society of Anesthesiologists physical status
PACU: Postanesthesia care unit
OR: Operating room
MT: Malignant tumor
FUSCC: Fudan University Shanghai Cancer Center
BMI: Body mass index
PRBC: Packed red blood cell
GA: General anesthesia
GEA: General anesthesia combined with epidural anesthesia
LOS: Length of stay
SD: Standard deviation
IQR: Interquartile range
ORs: Odds ratios
CI: Confidence interval
